# Publisher Correction: Fusion guide RNAs for orthogonal gene manipulation with Cas9 and Cpf1

**DOI:** 10.1038/s41467-017-02686-8

**Published:** 2018-01-16

**Authors:** Jiyeon Kweon, An-Hee Jang, Da-eun Kim, Jin Wook Yang, Mijung Yoon, Ha Rim Shin, Jin-Soo Kim, Yongsub Kim

**Affiliations:** 1 Department of Biomedical Science, University of Ulsan College of Medicine, Asan Medical Center, Seoul, 05505 Republic of Korea; 2 Department of Chemistry, Seoul National University, Seoul, 08826 Republic of Korea; 3 Center for Genome Engineering, Institute for Basic Science, Seoul, 08826 Republic of Korea

Correction to: *Nature Communications* 10.1038/s41467-017-01650-w, Article published online 23 November 2017

The originally published version of this Article contained an error in the spelling of the author Da-eun Kim, which was incorrectly given as Da-Eun Kim. Furthermore, in Figure 1a, the Cas9 protein was positioned incorrectly during typesetting. These errors have now been corrected in both the PDF and HTML versions of the Article.

