# Supplementary material for: Fusion guide RNAs for orthogonal gene manipulation with Cas9 and Cpf1
Source: Nat Commun. 2017 Nov 23;8:1723. doi: 10.1038/s41467-017-01650-w (PMC5700056; doi:10.1038/s41467-017-01650-w)
Supplement: Supplementary file 1 — Supplementary Information [file 41467_2017_1650_MOESM1_ESM.pdf]

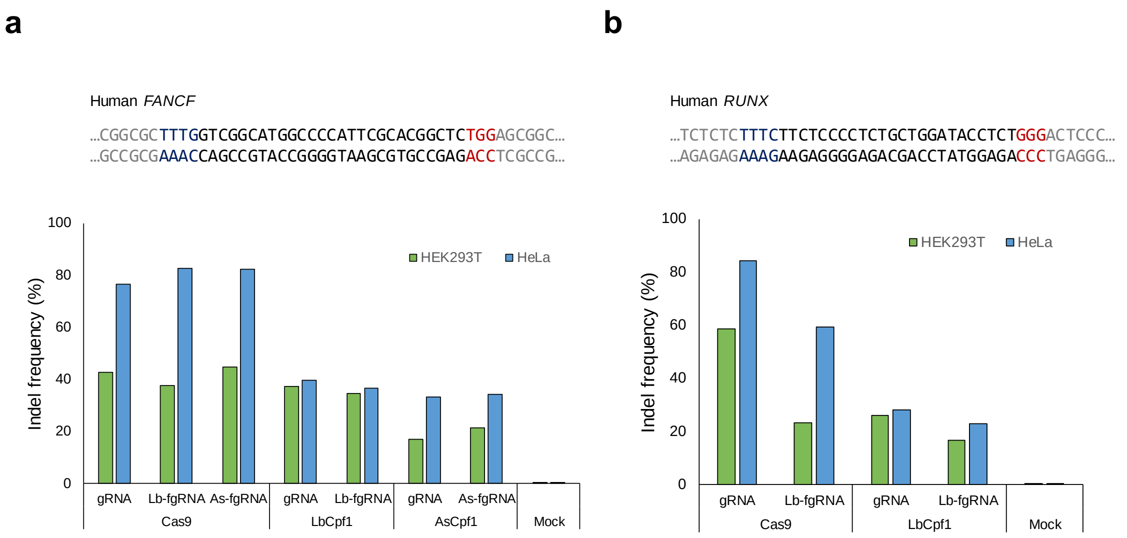

Supplementary Figure 1. Endogenous indel frequencies of fgRNAs with either 5′-scaffolds of LbCpf1 or AsCpf1 in two cell lines.

(a) fgRNAs containing the 5′-scaffold of LbCpf1 or AsCpf1 could function with Cas9, LbCpf1, or AsCpf1 to induce endogenous indels at the human *FANCF* locus. (b) fgRNAs targeting human *RUNX* gene could successfully induce indels in two different human cell lines, HEK293T and HeLa. Numerical data of targeted deep sequencing were shown in **Supplementary Table 3**.

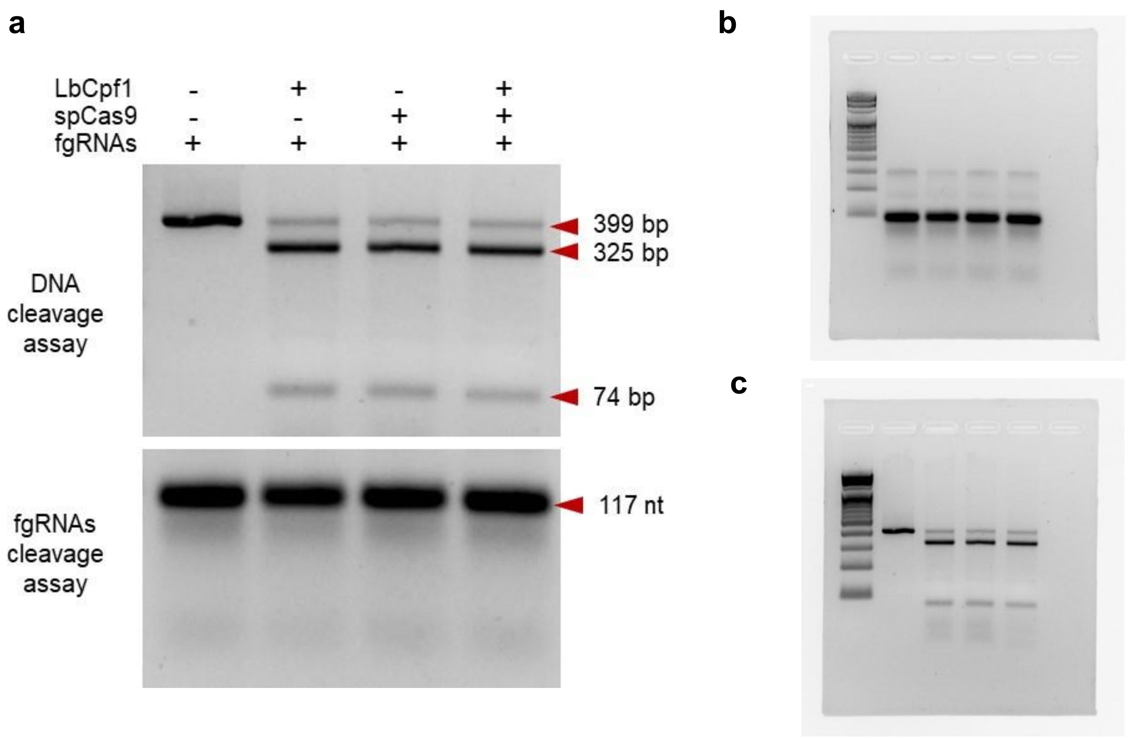

Supplementary Figure 2. *In vitro* RNA and DNA cleavage assay using Cas9 and Cpf1.

(a) Bottom, fgRNAs were not processed by LbCpf1 and spCas9 *in vitro*. 900nM fgRNAs were incubated with 300nM Cas proteins at 37°C for 30 min and analyzed by agarose gel electrophoresis. The lengths of *in vitro*-transcribed fgRNAs were 117 nucleotide (nt) as indicated by red arrow. Top, both LbCpf1 and spCas9 could work with fgRNAs to cleave target DNA *in vitro*. Same amount of fgRNAs and Cas proteins were incubated with 40nM of PCR amplified target DNA at 37°C for 30min. The lengths of PCR amplified target DNA were 399 bp, and estimated lengths of cleaved DNA fragments were 325bp and 74bp. Neither spCas9 nor LbCpf1 processed fgRNA but retained DNA cleavage activity *in vitro*. (b) Full gel image of fgRNAs cleavage assay. (c) Full gel image of DNA cleavage assay.

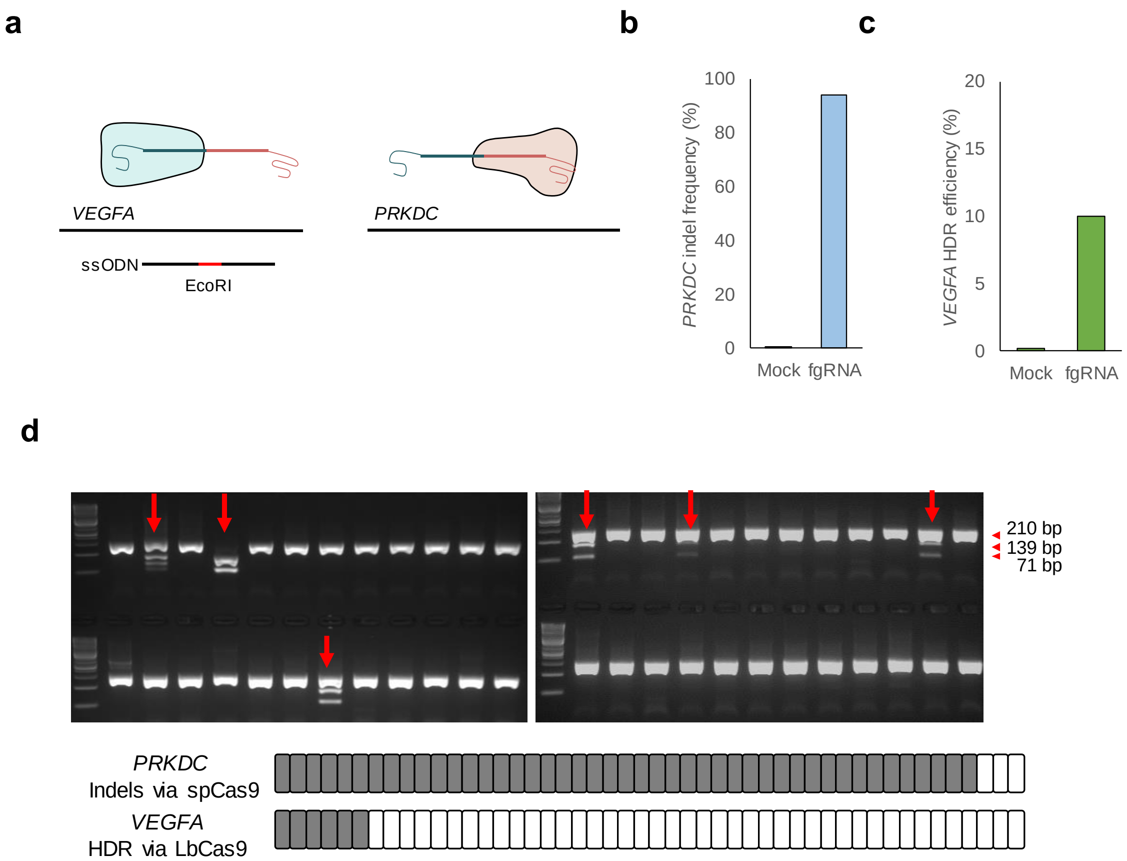

Supplementary Figure 3. Multiple genome editing using fgRNAs.

(a) Schematic overviews of fgRNAs-mediated multiple genome editing. fgRNAs containing target sequences of both *VEGFA* and *PRKDC* can induce HDR at the endogenous *VEGFA* locus with ssODN, similar to LbCpf1 and NHEJ at the endogenous *PRKDC* locus with spCas9. (b) fgRNA-mediated indel frequencies at the *PRKDC* locus were measured using targeted deep sequencing. Numerical data of targeted deep sequencing are shown in **Supplementary Table 3**. (c) fgRNA-mediated HDR frequencies at *VEGFA* locus were measured using targeted deep sequencing. (d) Single clone analysis to confirm whether HDR and NHEJ were induced in the same cells. Forty-five of 48 clones were identified to have disrupted alleles at the *PRKDC* locus using targeted deep sequencing. Six of 48 clones had HDR alleles at the *VEGFA* locus measured using EcoRI restriction enzyme digestion assay.

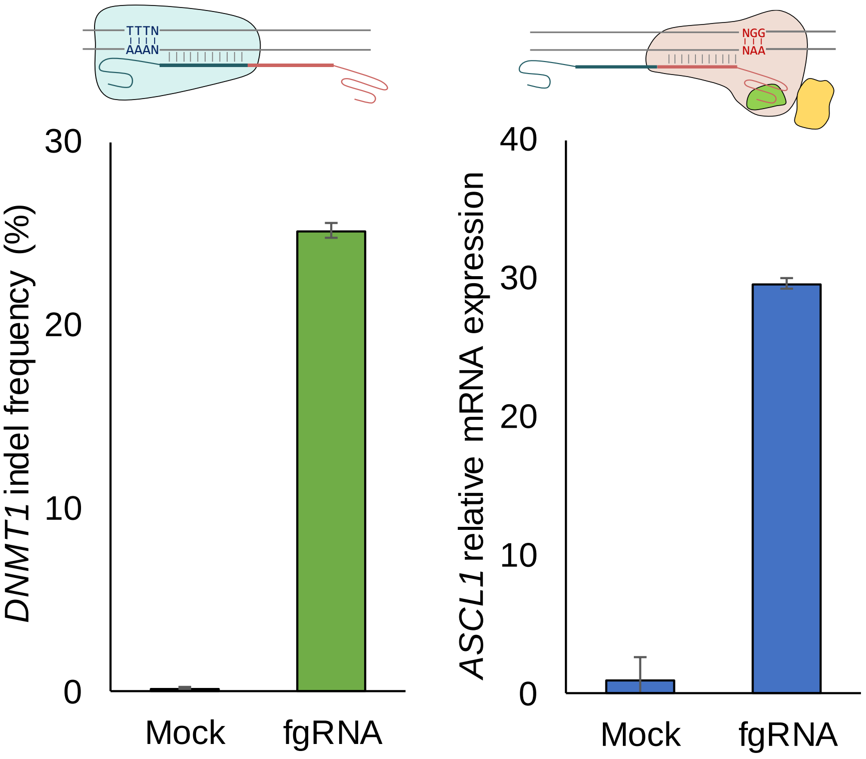

Supplementary Figure 4. Orthogonal gene manipulation using fgRNAs.
fgRNAs containing both *VEGFA* and *ASCL1* target sequences were co-transfected in HEK293T cells with LbCpf1, dCas9-VP64, and MS2-p65-HSF1. Indel frequencies at *VEGFA* locus were measured using targeted deep sequencing, and *ASCL* mRNA levels were measured using qPCR. Numerical data of targeted deep sequencing are shown in **Supplementary Table 3**. Error bars indicate s.e.m (n=3).

Supplementary Table 1. Target sequences of fgRNAs and guide RNAs used in this study.

Cas9 gRNA target sequences

Target gene	Target sequences (5' to 3' w/o PAM)
FANCF	tggccccattcgcacggctc
RUNX	cccctctgctggatacctct
VEGFA	ctaggaatattgaagggggc

Cpf1 gRNA target sequences

Target gene	Target sequences (5' to 3' w/o PAM)
FANCF	gtcggcatggccccattcgc
RUNX	ttctcccctctgctggatacctc
VEGFA	ctaggaatattgaagggggc
DNMT1_#3	ctgatggtccatgtctgttactc
DNMT1_#7	gctcagcaggcacctgcctcagc

Cpf1 gRNA target sequences

Target gene	Target sequences (5' to 3' w/o PAM)
FANCF	gtcggcatggccccattcgc
RUNX	ttctcccctctgctggatacctc
VEGFA	ctaggaatattgaagggggc
DNMT1_#3	ctgatggtccatgtctgttactc
DNMT1_#7	gctcagcaggcacctgcctcagc

fgRNA target sequences used in supplementary figure 3

Guide RNA name	Target sequences (5' to 3' w/o PAM)
Cpf1_fgRNA (+5)	ctaggaatattgaagggggcgtcgg
Cpf1_fgRNA (+10)	ctaggaatattgaagggggcgtcggcatgg
Cpf1_fgRNA (+15)	ctaggaatattgaagggggcgtcggcatggcccca
Cpf1_fgRNA (+20)	ctaggaatattgaagggggcgtcggcatggccccattcgc
Cas9_fgRNA (+5)	ttcgcctaggaatattgaagggggc
Cas9_fgRNA (+10)	ccccattcgcctaggaatattgaagggggc
Cas9_fgRNA (+15)	catggccccattcgcctaggaatattgaagggggc
Cas9_fgRNA (+20)	gtcggcatggccccattcgcctaggaatattgaagggggc

Supplementary Table 2. List of primers.

PCR primer sequences of on-target sites

Target gene	Primer name	Primer sequences (5' to 3')
FANCF	1st_F	cacctcatggaatcccttct
	1st_R	ccacaggctgctgagaaac
	Deep_F	ACACTCTTTCCCTACACGACGCTCTTCCGATCTctggcggtctcaagcactac
	Deep_R	GTGACTGGAGTTCAGACGTGTGCTCTTCCGATCTggcctggaagttcgctaatc
RUNX	1st_F	tttgggcctcataaacaacc
	1st_R	ggtgaaacaagctgccattt
	Deep_F	ACACTCTTTCCCTACACGACGCTCTTCCGATCTgagcatcaccaacccacag
	Deep_R	GTGACTGGAGTTCAGACGTGTGCTCTTCCGATCTgggggactcaatgatttctt
VEGFA	1st_F	gaagcaactccagtcccaaa
	1st_R	ccaaggttcacagcctgaaa
	Deep_F	ACACTCTTTCCCTACACGACGCTCTTCCGATCTgtgggtgagtgagtgtgtgc
	Deep_R	GTGACTGGAGTTCAGACGTGTGCTCTTCCGATCTagggagcaggaaagtgaggt
DNMT1	#3_1st_F	GCCACAAACACCATGTACCA
	#3_1st_R	ATCTTTCTCAAGGGGCTGCT
	#3_Deep_F	ACACTCTTTCCCTACACGACGCTCTTCCGATCTtccttagcagcttcctcctc
	#3_Deep_R	GTGACTGGAGTTCAGACGTGTGCTCTTCCGATCTtcacttgagcctctgggtct
	#7_1st_F	AAGTCACTCTGGGGAACACG
	#7_1st_R	CTTTGTATGTTGGCCAAAGC
	#7_Deep_F	ACACTCTTTCCCTACACGACGCTCTTCCGATCTgcctgcagaagtgagtcttg
	#7_Deep_R	GTGACTGGAGTTCAGACGTGTGCTCTTCCGATCTgctaccattcctaggcagca
TREX2	1st_F	tcgggagatgctatctgctt
	1st_R	gggactcctcttggtcacct
	Deep_F	ACACTCTTTCCCTACACGACGCTCTTCCGATCTtaccgtgcacctaacagtgg
	Deep_R	GTGACTGGAGTTCAGACGTGTGCTCTTCCGATCTgaaggacctggtgcccttt
PRKDC	1st_F	ggaaggcctggccagtaag
	1st_R	tatcccttaggcgtttttgg
	Deep_F	ACACTCTTTCCCTACACGACGCTCTTCCGATCTccgaatcaacatggaaacct
	Deep_R	GTGACTGGAGTTCAGACGTGTGCTCTTCCGATCTcgcctcagcttcctaaagtg

PCR primer sequences of off-target sites

On-target sites	Primer name	Primer sequences (5' to 3')
DNMT1_#3	OT1_1st_F	gcgggatgtccttttctacc
	OT1_1st_R	tgatgggaaagtgtgcaaat
	OT1_Deep_F	ACACTCTTTCCCTACACGACGCTCTTCCGATCT ctttcccatcccggaagtat
	OT1_Deep_R	GTGACTGGAGTTCAGACGTGTGCTCTTCCGATCTtagcatggaggagaggcaat
	OT2_1st_F	tgtccaatgccttccttacc
	OT2_1st_R	agtcccatgaggaggtccag
	OT2_Deep_F	ACACTCTTTCCCTACACGACGCTCTTCCGATCTttcccttagggtggaatgtg
	OT2_Deep_R	GTGACTGGAGTTCAGACGTGTGCTCTTCCGATCTaggactctacccccaccact
	OT3_1st_F	agagagggtcaagtgctcca
	OT3_1st_R	ccttggattctcatcccta
	OT3_Deep_F	ACACTCTTTCCCTACACGACGCTCTTCCGATCTcccttttccgatggagtgta
	OT3_Deep_R	GTGACTGGAGTTCAGACGTGTGCTCTTCCGATCTgctcttcccctcaaccacta
DNMT1_#7	OT1_1st_F	aagccctttccagagctagg
	OT1_1st_R	ggagcaaggtggctgagtag
	OT1_Deep_F	ACACTCTTTCCCTACACGACGCTCTTCCGATCTgcctgcagaagtgagtcttg
	OT1_Deep_R	GTGACTGGAGTTCAGACGTGTGCTCTTCCGATCTgctaccattcctaggcagca

PCR primer sequences of qRT-PCR

Target gene	Primer name	Primer sequences (5' to 3')
MYOD	qRT-1F	cgccatccgctatatcgagg
	qRT-1R	ctgtagtccatcatgccgtcg
	qRT-2F	cggcatgatggactacagcg
	qRT-2R	caggcagtctaggctcgac
ASCL	qRT-1F	cccaagcaagtcaagcgaca
	qRT-1R	aagccgctgaagttgagcc
	qRT-2F	cgcggccaacaagaagatg
	qRT-2R	cgacgagtaggatgagaccg

Supplementary Table 3. Indel frequencies measured by targeted deep sequencing.

Target sites	Cas proteins	Guide RNAs	Cell types	Indel frequency (%)	Related figures
VEGFA	spCas9	spCas9 gRNAs	HeLa	95.5	Figure 1b
		LbCpf1 gRNAs	HeLa	0.2	
		fgRNAs	HeLa	86.9	
	LbCpf1	spCas9 gRNAs	HeLa	0.2	
		LbCpf1 gRNAs	HeLa	70.6	
		fgRNAs	HeLa	55.0	
	Mock	Mock	HeLa	0.2	
FANCF	LbCpf1	LbCpf1 gRNAs	HeLa	25.7	Figure 1c
		fgRNAs	HeLa	21.0	
		Mock	HeLa	0.1	
DNMT1_#3		LbCpf1 gRNAs	HeLa	48.6	
		fgRNAs	HeLa	51.4	
		Mock	HeLa	0.1	
DNMT1_#7		LbCpf1 gRNAs	HeLa	36.9	
		fgRNAs	HeLa	34.3	
		Mock	HeLa	0.1	
RUNX		LbCpf1 gRNAs	HeLa	39.6	
		fgRNAs	HeLa	36.4	
		Mock	HeLa	0.1	
FANCF	spCas9	LbCpf1 gRNAs	HeLa	73.4	Figure 1d
		fgRNAs	HeLa	57.7	
		Mock	HeLa	0.1	
RUNX		LbCpf1 gRNAs	HeLa	76.5	
		fgRNAs	HeLa	82.7	
		Mock	HeLa	0.1	
VEGFA	spCas9	Matched spCas9 gRNAs	HeLa	93.3	Figure 2a
		Mismatched_1_spCas9 gRNAs	HeLa	96.2	
		Mismatched_3_spCas9 gRNAs	HeLa	76.5	
		Mismatched_5_spCas9 gRNAs	HeLa	64.4	
		Mismatched_7_spCas9 gRNAs	HeLa	92.9	
		Mismatched_9_spCas9 gRNAs	HeLa	31.0	
		Mismatched_11_spCas9 gRNAs	HeLa	87.8	
		Mismatched_13_spCas9 gRNAs	HeLa	94.2	
		Mismatched_15_spCas9 gRNAs	HeLa	94.2	
		Mismatched_17_spCas9 gRNAs	HeLa	52.4	
		Mismatched_19_spCas9 gRNAs	HeLa	75.9	
		Matched fgRNAs	HeLa	86.4	
	spCas9	Mismatched_1_fgRNAs	HeLa	87.8	
		Mismatched_3_fgRNAs	HeLa	62.1	
		Mismatched_5_fgRNAs	HeLa	66.9	
		Mismatched_7_fgRNAs	HeLa	75.6	
		Mismatched_9_fgRNAs	HeLa	64.9	
		Mismatched_11_fgRNAs	HeLa	76.2	
		Mismatched_13_fgRNAs	HeLa	85.3	
		Mismatched_15_fgRNAs	HeLa	82.9	
		Mismatched_17_fgRNAs	HeLa	41.1	
		Mismatched_19_fgRNAs	HeLa	28.6	

Target sites	Cas proteins	Guide RNAs	Cell types	Indel frequency (%)	Related figures
VEGFA	LbCpf1	Matched LbCpf1 gRNAs	HeLa	48.3	Figure 2a
		Mismatched_1_LbCpf1 gRNAs	HeLa	16.2	
		Mismatched_3_LbCpf1 gRNAs	HeLa	33.3	
		Mismatched_5_LbCpf1 gRNAs	HeLa	17.1	
		Mismatched_7_LbCpf1 gRNAs	HeLa	34.3	
		Mismatched_9_LbCpf1 gRNAs	HeLa	37.0	
		Mismatched_11_LbCpf1 gRNAs	HeLa	24.6	
		Mismatched_13_LbCpf1 gRNAs	HeLa	32.1	
		Mismatched_15_LbCpf1 gRNAs	HeLa	28.7	
		Mismatched_17_LbCpf1 gRNAs	HeLa	21.9	
		Mismatched_19_LbCpf1 gRNAs	HeLa	45.3	
		Matched fgRNAs	HeLa	33.2	
		Mismatched_1_fgRNAs	HeLa	11.6	
		Mismatched_3_fgRNAs	HeLa	21.4	
		Mismatched_5_fgRNAs	HeLa	7.7	
		Mismatched_7_fgRNAs	HeLa	21.2	
		Mismatched_9_fgRNAs	HeLa	26.8	
		Mismatched_11_fgRNAs	HeLa	15.4	
		Mismatched_13_fgRNAs	HeLa	16.3	
		Mismatched_15_fgRNAs	HeLa	12.0	
		Mismatched_17_fgRNAs	HeLa	20.0	
		Mismatched_19_fgRNAs	HeLa	35.8	
DNMT_#3_ON	LbCpf1	LbCpf1 gRNAs	HeLa	48.6	Figure 2b
		fgRNAs	HeLa	51.4	
		Mock	HeLa	0.15	
DNMT_#3_OT1		LbCpf1 gRNAs	HeLa	2.4	
		fgRNAs	HeLa	2.1	
		Mock	HeLa	0.15	
DNMT_#3_OT2		LbCpf1 gRNAs	HeLa	0.4	
		fgRNAs	HeLa	0.3	
		Mock	HeLa	0.15	
DNMT_#3_OT3		LbCpf1 gRNAs	HeLa	0.3	
		fgRNAs	HeLa	0.2	
		Mock	HeLa	0.2	
DNMT_#7_ON		LbCpf1 gRNAs	HeLa	36.9	
		fgRNAs	HeLa	34.3	
		Mock	HeLa	0.15	
DNMT_#7_OT1		LbCpf1 gRNAs	HeLa	2.1	
		fgRNAs	HeLa	1.7	
		Mock	HeLa	0.15	
VEGFA	spCas9	fgRNA(+20)	HeLa	74.2	Figure 3
		fgRNA(+15)	HeLa	75.8	
		fgRNA(+10)	HeLa	70.7	
		fgRNA(+5)	HeLa	70.5	
		fgRNA(+0)	HeLa	68.6	
	LbCpf1	fgRNA(+20)	HeLa	22.5	
		fgRNA(+15)	HeLa	21.9	
		fgRNA(+10)	HeLa	28.8	
		fgRNA(+5)	HeLa	28.3	
		fgRNA(+0)	HeLa	25.9	

Target sites	Cas proteins	Guide RNAs	Cell types	Indel frequency (%)	Related figures
VEGFA	spCas9	fgRNAs	HeLa	1.6	Figure 4b
	LbCpf1	fgRNAs	HeLa	40.7	
	spCas9+LbCpf1	fgRNAs	HeLa	24.3	
	Mock	Mock	HeLa	1.6	
FANCF	spCas9	fgRNAs	HeLa	61.9	
	LbCpf1	fgRNAs	HeLa	0.1	
	spCas9+LbCpf1	fgRNAs	HeLa	57.3	
	Mock	Mock	HeLa	0.1	
VEGFA	spCas9	fgRNAs	HeLa	2.8	Figure 4c
	LbCpf1	fgRNAs	HeLa	49.5	
	spCas9+LbCpf1	fgRNAs	HeLa	29.9	
	Mock	Mock	HeLa	1.6	
TREX2	spCas9	fgRNAs	HeLa	67.1	
	LbCpf1	fgRNAs	HeLa	0.3	
	spCas9+LbCpf1	fgRNAs	HeLa	64.1	
	Mock	Mock	HeLa	0.3	
VEGFA	spCas9+LbCpf1	fgRNAs	HeLa	28.8	Figure 4d
RUNX	spCas9	spCas9 gRNAs	HEK293T	42.5	Supplementary figure 1a
		LbCpf1 fgRNAs	HEK293T	37.6	
		AsCpf1 fgRNAs	HEK293T	44.6	
	LbCpf1	LbCpf1 gRNAs	HEK293T	37.0	
		LbCpf1 fgRNAs	HEK293T	34.3	
	AsCpf1	AsCpf1 gRNAs	HEK293T	16.9	
		AsCpf1 fgRNAs	HEK293T	21.2	
	Mock	Mock	HEK293T	0.3	
	spCas9	spCas9 gRNAs	HeLa	76.5	
		LbCpf1 fgRNAs	HeLa	82.7	
		AsCpf1 fgRNAs	HeLa	82.1	
	LbCpf1	LbCpf1 gRNAs	HeLa	39.6	
		LbCpf1 fgRNAs	HeLa	36.4	
	AsCpf1	AsCpf1 gRNAs	HeLa	33.1	
		AsCpf1 fgRNAs	HeLa	34.0	
	Mock	Mock	HeLa	0.1	
FANCF	spCas9	spCas9 gRNAs	HEK293T	58.4	Supplementary figure 1b
		LbCpf1 fgRNAs	HEK293T	23.3	
	LbCpf1	LbCpf1 gRNAs	HEK293T	26	
		LbCpf1 fgRNAs	HEK293T	16.6	
	Mock	Mock	HEK293T	0.1	
	spCas9	spCas9 gRNAs	HeLa	84.2	
		LbCpf1 fgRNAs	HeLa	59.1	
	LbCpf1	LbCpf1 gRNAs	HeLa	28.1	
		LbCpf1 fgRNAs	HeLa	22.7	
	Mock	Mock	HeLa	0.1	
PRKDC	spCas9	fgRNAs	HeLa	82.4	Supplementary figure 3
DNMT1	LbCpf1	fgRNAs	HeLa	25.2	Supplementary figure 4
